# Determinants of Prevalent HIV Infection and Late HIV Diagnosis among Young Women with Two or More Sexual Partners in Beira, Mozambique

**DOI:** 10.1371/journal.pone.0063427

**Published:** 2013-05-17

**Authors:** Arlinda Zango, Karine Dubé, Sílvia Kelbert, Ivete Meque, Fidelina Cumbe, Pai Lien Chen, Josefo J. Ferro, Paul J. Feldblum, Janneke van de Wijgert

**Affiliations:** 1 Universidade Católica de Moçambique/Catholic University of Mozambique, Centro de Investigação de Doenças Infecciosas (UCM)/Center for Infectious Disease Research (CIDI), Beira, Mozambique; 2 Clinical Sciences Department, FHI 360, Durham, North Carolina, United States of America; 3 Clinical Operations Office, United States Military HIV Research Program (MHRP), Henry M. Jackson Foundation for the Advancement of Military Medicine, Inc. (HJF), Bethesda, Maryland, United States of America; 4 London School of Hygiene and Tropical Medicine, London, United Kingdom; 5 Amsterdam Institute for Global Health and Development (AIGHD) and Academic Medical Center of the University of Amsterdam, Amsterdam, The Netherlands; 6 University of Liverpool, Institute of Infection and Global Health, Liverpool, United Kingdom; Vanderbilt University, United States of America

## Abstract

**Background:**

The prevalence and determinants of HIV and late diagnosis of HIV in young women in Beira, Mozambique, were estimated in preparation for HIV prevention trials.

**Methods:**

An HIV prevalence survey was conducted between December 2009 and October 2012 among 1,018 women aged 18–35 with two or more sexual partners in the last month. Participants were recruited in places thought by recruitment officers to be frequented by women at higher-risk, such as kiosks, markets, night schools, and bars. Women attended the research center and underwent a face-to-face interview, HIV counseling and testing, pregnancy testing, and blood sample collection.

**Results:**

HIV prevalence was 32.6% (95% confidence interval (CI) 29.7%–35.5%). Factors associated with being HIV infected in the multivariable analysis were older age (p<0.001), lower educational level (p<0.001), self-reported genital symptoms in the last 3 months (adjusted odds ratio (aOR) = 1.4; CI 1.1–2.0), more than one lifetime HIV test (aOR = 0.4; CI 0.3–0.6), and not knowing whether the primary partner has ever been tested for HIV (aOR = 1.7; CI 1.1–2.5). About a third (32.3%) of participants who tested HIV-positive had a CD4 lymphocyte count of <350 cells/µl at diagnosis. Factors associated with late diagnosis in multivariable analyses were: not knowing whether the primary partner has ever been tested for HIV (aOR = 2.2; CI 1.1–4.2) and having had a gynecological pathology in the last year (aOR = 3.7; CI 1.2–12.0).

**Conclusions:**

HIV prevalence and late diagnosis of HIV infection were high in our study population of young women with sexual risk behavior in Beira, Mozambique. HIV prevention programs should be strengthened, health care providers should be sensitized, and regular HIV testing should be encouraged to enroll people living with HIV into care and treatment programs sooner.

## Introduction

Despite recent progress in HIV prevention, diagnosis and treatment in most regions of the world, the control of the HIV/AIDS epidemic remains a challenge. In Sub-Saharan Africa, an estimated 23.1 million people were living with HIV at the end of 2010 [1]. Mozambique is among the 10 highest-prevalence countries in the world, with an estimated 1.4 million people living with HIV at the end of 2009 [2]. A national population-based survey completed in 2009 showed an 11.5% national prevalence [3], with marked differences across geographical areas: the southern region had the highest HIV-prevalence at 21%, followed by the central and northern regions at 18% and 9.1%, respectively. Overall HIV prevalence was higher among women than men (13.1% vs. 9.1%) [3]. The HIV epidemic in Mozambique took hold while the country was economically impoverished due to 16 years of civil war, and HIV spread continues unabated because of high levels of poverty and gender inequality, a poor average educational level, and a health care system that cannot cope [4].

Local epidemiological data are needed to assess the evolution of the epidemic, to maximize efficacy of HIV prevention programs, and to plan for optimal use of limited resources. In addition, Mozambique is interested in hosting HIV prevention intervention trials, and data on HIV prevalence and incidence are needed to properly power these trials. Recent HIV prevention trials in neighboring countries have showed promising results for male circumcision, oral pre-exposure prophylaxis and topical microbicides with antiretroviral drugs [5]–[10]. Efforts are underway to test new HIV vaccines and vaccination strategies in Southern Africa, including Mozambique, starting in 2015 [11].

Mozambique’s participation in such HIV prevention trials will require study populations with high HIV incidence as well as research centers with an adequate clinical trial infrastructure and community linkages [12]. From 2008–2012, researchers from the Catholic University of Mozambique (UCM), FHI 360 and the Amsterdam Institute for Global Health and Development (AIGHD) collaborated to establish a new clinical research center in Beira, Mozambique: the Centro de Investigação de Doenças Infecciosas or Center for Infectious Disease Research (CIDI). This capacity-building initiative was part of two larger international networks aimed at improving HIV prevention trial capacity in sub-Saharan Africa: the Site Identification and Development Initiative (SIDI) funded by the U.S. Agency for International Development (USAID), and the African-European HIV Vaccine Development Network (AfrEVacc) funded by the European and Developing Country Clinical Trials Partnership (EDCTP). We collaborated on a study to measure HIV prevalence and the determinants of prevalent HIV infection and late HIV diagnosis among young women with two or more sexual partners in Beira, Mozambique.

## Materials and Methods

### Ethical Review and Approval

The study was approved by the Comité National de Bioética para a Saúde/National Committee for Bioethics in Maputo, Mozambique, the FHI 360 Protection of Human Subjects Committee, and the Division of Human Subjects Protection of the Walter Reed Army Institute of Research. Participants received approximately 5 USD reimbursement per scheduled study visit.

### Study Design and Population

Beira is a port city in Sofala Province in central Mozambique, connected to Malawi and Zimbabwe by major commercial transit routes. It is a diverse and vibrant city, with a population of about 430,000 persons (2007 census), where multiple cultural and ethnic groups converge. Beira’s five administrative areas are Chiveve, Munhava, Manga Loforte, Inhamizua and Nhangau. Our study population included women from all five areas. We did not set out to create a representative sample of women in Beira; instead, we recruited and enrolled a sizable group of women at higher risk for HIV infection, defined as having had two or more sexual partners in the previous month. Recruitment took place at locations where outreach workers expected to find young women at higher risk for HIV acquisition, such as kiosks, markets, night schools, bars, certain streets, and parking areas for long-haul truck drivers. The recruitment team also collaborated with community activists who distribute condoms to female sex workers. Volunteer visited the above-mentioned recruitment areas daily and recruitment occurred using a pre-approved recruitment script. Recruitment also took place in targeted residential areas two or three times per month during presentations by a local drama group to educate communities about HIV and to raise interest in the study. These performances were used both to recruit study participants (in groups) and to address possible rumors about the study.

The majority of the study candidates were offered transportation the first time they came to the center so that they would know its location. Some study candidates visited the research center on their own, having heard about the study through relatives and friends or through the drama group performances.

The study was conducted between 2009 and 2012 in three phases: 1) a cross-sectional survey to assess HIV prevalence and its determinants and to estimate HIV incidence using the BED-CEIA assay; 2) a prospective cohort study to measure HIV incidence directly; and 3) a BED False Recent phase to obtain a locally-derived false recent rate to adjust the cross-sectional HIV incidence estimate. This paper summarizes the results from the cross-sectional survey only.

### Study Procedures

Women were eligible for study participation if they were 18–35 years, had Mozambican citizenship, had been sexually active with at least two sexual partners in the previous month, did not know their HIV status or their last HIV test result (within the last 6 months) was negative, did not have a history of antiretroviral treatment (ART) or non-therapeutic injection drug use, were not currently enrolled in another HIV-related study, and were willing and able to participate in the study and provide informed consent.

All study procedures were conducted at the CIDI research center by trained study staff in private rooms. After written informed consent was obtained and eligibility confirmed, each participant underwent a face-to-face interview; received counseling about HIV, other sexually transmitted infections (STI) and family planning; and gave blood and urine samples. Rapid HIV testing was performed on one of the blood samples in the presence of the study participant immediately following collection. Each sample was tested with the Determine HIV-1/2 test (Alere Medical Co. Ltd., Chiba, Japan) and the Uni-Gold HIV test (HIV Trinity BioTech PLC, Bray, Ireland). The SD Bioline HIV-1/2 version 3.0 test (Standard Diagnostics Inc, Kyonggi-do, Korea) was used as a tie-breaker when needed. In addition, a rapid urine hCG pregnancy test (Healthease Preg n Care, NEOMED IPA, Tzaneen, South Africa) was done on the urine sample. The results of the Determine, Uni-Gold and pregnancy tests were given to the participant during the study visit. HIV-positive women were asked to return to the research center to obtain their CD4 count results and were referred to local health centers for further evaluation and care. Women with unclear HIV results were asked to return to the research center to receive the results of additional HIV testing (Vironostika HIV-1 Uni-Form II Plus 0 test, BioMerieux BV, Boxtel, Netherlands). Pregnant women were referred to antenatal care.

Immediately following diagnosis, one EDTA blood sample from each HIV-positive woman was sent to a local laboratory (Centro de Saúde Urbano da Ponta-Gêa, Beira) for CD4 lymphocyte count determination using flow cytometry (BD FACSCalibur, San Jose, CA, USA). The remaining blood samples were sent to the study laboratory at UCM for processing and storage at −80°C.

### Statistical Analysis

All data were recorded on standardized case report forms (CRFs), which were double-entered into a Clintrial database (Oracle Health Sciences, Redwood Shores, CA, USA) and transmitted to the FHI 360 server using 21 Code of Federal Regulations Part 11-compliant Citrix 12.3 software. Data were analyzed using STATA software version 12.0 (Statacorp, College Station, TX, USA).

Descriptive statistics were used to summarize baseline demographic, behavioral and clinical characteristics. Categorical variables were expressed as percentages, and continuous variables as medians with inter-quartile ranges (IQR). We used bivariable logistic regression models to assess predictors of prevalent HIV infection one at a time. All factors associated with HIV-1 infection at p≤0.25 in the bivariable analyses were considered for inclusion in an explanatory multivariable logistic regression model using a forward stepwise selection (with a cut-off of p≤0.05 to be retained in the model). Predictors were added to the multivariable model in three batches according to a hierarchical framework: demographic factors, sexual and behavioral factors, and clinical factors (Tables 1 and 2). When two exposure variables were highly correlated, one was chosen for inclusion in the final model to avoid co-linearity. However, self-reported sex work and receiving money or goods in exchange for sex were each considered as separate predictors because they are considered socially distinct situations in Mozambique. A similar approach was used for models with late HIV diagnosis as the outcome (defined as having a CD4 lymphocyte count of ≤350cells/µL at the time of diagnosis). The final multivariable models contained all factors that remained significant at p≤0.05.

**Table 1:** Demographic factors and bivariable associations with prevalent HIV infection.

Characteristics	Total (%) n = 1018	HIV+ (%) n = 332 (32.6)	Crude OR* (95% CI)	*P-value* *
**Age, years (median = 22; IQR = 19–25)**				
18–19	277 (27.2)	48 (17.3)	1.0	
20–21	205 (20.1)	50 (24.4)	1.54 (0.98–2.41)	
22–23	165 (16.2)	48 (29.1)	1.96 (1.23-3-11)	<0.001§
24–26	177 (17.4)	78 (44.1)	3.76 (2.40–5.89)	
27–35	194 (19.1)	108 (55.7)	5.99 (3.78–9.49)	
**Area of living** 2				
Chiveve	587 (57.8)	178 (30.3)	1.0	
Nhamisua	291 (28.6)	105 (36.1)	1.30 (0.96–1.75)	0.169
Munhava, Manga Loforte, Nhangau	138 (13.6)	49 (35.5)	1.27 (0.86–1.87)	
**Marital status** 2				
Single	821 (80.8)	267 (32.5)	1.0	
Married	181 (17.8)	57 (31.5)	0.95 (0.67–1.35)	0.140
Divorced/Widowed	14 (1.4)	8 (57.1)	2.77 (0.95–8.08)	
**Level of education**				
None/Primary	149 (14.6)	77 (51.7)	1.0	
Incomplete Secondary	591 (58.1)	199 (33.7)	0.47 (0.33–0.69)	<0.001§
Secondary and above	278 (27.3)	56 (20.1)	0.24 (0.15–0.37)	
**Occupation**				
Unemployed	371 (36.4)	128 (34.5)	1.0	
Student	292 (28.7)	65 (22.3)	0.54 (0.38–0.77)	
Self-reported sex worker	35 (3.4)	16 (45.7)	1.60 (0.79–3.22)	<0.001
Sales	201 (19.7)	75 (37.3)	1.13 (0.79–1.62)	
Other^4^	119 (11.7)	48 (40.3)	1.28 (0.84–1.96)	
**Monthly income, $USD** 1,5				
No income	675 (66.4)	201 (29.8)	1.0	
Lower (up to $40)	169 (16.6)	59 (34.9)	1.26 (0.89–1.81)	0.004§
Higher ($41 to $1000)	173 (17.0)	71 (41.0)	1.64 (1.16–2.32)	
**Number of pregnancies** 1				
0	272 (26.8)	59 (21.6)	1.0	
1	280 (27.5)	86 (30.7)	1.61 (1.09–2.37)	<0.001§
2 or more	465 (45.7)	187 (40.2)	2.43 (1.72–3.42)	
**Family planning use**				
None	693 (68.0)	230 (33.2)	1.0	
Hormonal contraceptives^5^	231 (22.7)	83 (35.9)	1.13 (0.83–1.54)	
Condoms	81 (7.9)	17 (21.0)	0.53 (0.31–0.94)	0.046
Other (traditional)	13 (1.28)	2 (15.4)	0.36 (0.08–1.67)	
**Alcohol on ≥3days/week**				
No consumption	923 (90.7)	295 (31.9)	1.0	
At least 1 drink (1–4)	95 (9.3)	37 (38.9)	1.36 (0.88–2.09)	0.166

IQR: Inter-quartiles Range (25%–75%). CI: Confidence interval.

* Crude OR and P-value for the association between each variable and HIV-1 infection (chi-square test).

§ P-value from chi-square test for trend.

1 Missing data = 1.

2 Missing data = 2.

3 Missing data = 3.

4 Includes office work, manual labor, food service and housewife.

5 Local currency (Metical): 1 USD = 25 Meticais in November 2010.

5 Hormonal contraceptives: oral contraceptives, injectables, implants.

**Table 2:** Behavioral and clinical factors and bi-variable associations with prevalent HIV infection.

Characteristics	Total (% of total) n = 1018	HIV+ (% of group) n = 332 (32.6)	Crude Odds Ratio* (95% CI)	*p-value* *
**Payment for sex in last 30 days** 1				
No	830 (81.6)	262 (31.6)	1.0	
Yes	187 (18.4)	69 (36.9)	1.27 (0.91–1.77)	0.160
**VS partners in last 30 days** 1 **(median = 2; IQR = 2–3)**				
2	692 (68.0)	203 (29.3)	1.0	
3	252 (24.8)	93 (36.9)	1.41 (1.04–1.91)	<0.001§
4–16	73 (7.2)	35 (47.9)	2.22 (1.36–3.63)	
**# VS in last 30 days with PP** 1 **(median = 5; IQR = 2–9)**				
0–2	273 (26.8)	83 (30.4)	1.0	
3–4	212 (20.9)	66 (31.1)	1.03 (0.70–1.53)	
5–6	145 (14.3)	42 (28.9)	0.93 (0.60–1.45)	0.103§
7–60	387 (38.0)	141 (36.4)	1.31 (0.94–1.83)	
**# VS in last 30 days with CP (median = 3; IQR = 2–5)**				
0–2	468 (45.9)	130 (27.8)	1.0	
3–4	243 (23.9)	79 (32.5)	1.25 (0.89–1.75)	
5–6	116 (11.4)	40 (34.5)	1.37 (0.89–2.11)	<0.001§
7–60	191 (18.8)	83 (43.5)	1.99 (1.40–2.85)	
**Use of condom during VS with PP** 3,4				
Never/rarely	735 (79.8)	246 (33.5)	1.0	
Half of the times/always	186 (20.2)	47 (25.3)	0.67 (0.47–0.97)	0.032
**Use of condom during VS with CP** 1				
Never/rarely	590 (58.0)	205 (34.8)	1.0	
Half of the times/always	427 (41.9)	127 (29.7)	0.97 (0.61–1.04)	0.093
**VS last 30 days with any HIV+ partner**				
No	261 (25.6)	72 (27.6)	1.0	
Do not know	757 (74.4)	260 (34.4)	1.37 (1.01–1.87)	0.044
**PP has had sex with CP in the last 30 days** 1,4				
No	267 (29.2)	78 (29.2)	1.0	
Yes	650 (70.9)	215 (33.1)	1.20 (0.88–1.63)	0.254
**Self-reported STI episodes**				
No	976 (95.9)	311 (31.7)	1.0	
Yes	42 (4.1)	21 (50.0)	2.14 (1.15–3.98)	0.014
**Self-reported genital symptoms last 3 months**				
None	451 (44.3)	132 (29.3)	1.0	
At least 1 symptom	567 (55.7)	200 (35.3)	1.32 (1.01–1.72)	0.042
**Self-reported UTI episodes last year**				
None	890 (87.4)	281 (31.6)	1.0	
1	91 (96.4)	35 (38.5)	1.35 (0.87–2.12)	0.053§
2–12	37 (3.6)	16 (43.2)	1.65 (0.85–3.22)	
**HIV tests in lifetime** 1 **(median = 1; IQR = 0–1)**				
Never	442 (43.6)	153 (34.5)	1.0	
Only one	334 (32.8)	125 (37.4)	1.14 (0.84–1.52)	0.005§
More than one (2–20)	240 (23.6)	54 (22.5)	0.55 (0.38–0.79)	
**Primary partner ever tested for HIV** 3,4				
No	234 (25.5)	81 (34.6)	1.0	
Yes	299 (32.6)	65 (21.7)	0.52 (0.36–0.77)	<0.001
Do not know	385 (41.9)	148 (38.4)	1.18 (0.84–1.66)	
**Occupational exposure to HIV** 3				
No	815 (80.1)	243 (29.8)	1.0	
Yes	42 (4.1)	18 (42.9)	1.77 (0.94-3-32)	0.002
Do not know	158 (15.5)	70 (44.3)	1.87 (1.32–2.66)	
**Gynecological pathologies**				
No	995 (97.7)	318 (31.9)	1.0	
Yes	23 (2.3)	14 (60.9)	3.31 (1.41–7.76)	0.003

IQR: Inter-quartile range (25%–75%). CI: Confidence interval. VS: vaginal sex; PP: primary partner; CP: casual partner. STI: sexually transmitted infection. UTI: urinary tract infection.

* Crude Odds Ratio and p-value for the association between each variable and HIV-1 infection (chi-square test).

§ p-value from chi-square test for trend.

1 Missing data = 1.

2 Missing data = 2.

3 Missing data = 3.

4 Not applicable (no PP): use of condom during VS with PP = 94; PP have had sex with CP = 100; PP ever tested for HIV = 97.

## Results

### Disposition and Baseline Characteristics

A total of 1,070 women were recruited from which 1,018 were enrolled in the cross-sectional survey. The main reason for ineligibility was having reported fewer than two sexual partners in the last month. One participant withdrew early due to unwillingness to provide a blood sample.

The median age of study participants was 22 years (Table 1). Most participants (80.8%) were single, had incomplete secondary school education (58.1%) and had no income (66.4%). Only 3.4% of the women reported being a sex worker but 18.4% reported having received money or goods as payment for sex in the month prior to joining the study. Most women had at least one child (73.2%) and were not using any family planning (68.0%); 8.1% tested positive on the urine pregnancy test at baseline. Few women (9.3%) reported one or more alcoholic drinks on three or more days per week. The median number of sex partners in the month prior to study participation was two, with 32.0% of the women reporting three or more partners (Table 2). Most women reported never or rarely using a condom during vaginal sex with their primary partners (79.8%) and/or casual partners (58.0%), respectively. Self-reported anal sex prevalence was low at 1.5%. More than half of the women (55.7%) reported at least one genital symptom in the last 3 months, 2.3% reported a history of gynecological pathologies in the last year, and 8.8% had ever had a blood transfusion. Almost half of the participants (43.6%) had never been tested for HIV, and 23.6% had had more than one HIV test in their lifetime.

### HIV Prevalence and Determinants of Prevalent HIV

The HIV prevalence in this study population was 32.6% (95% confidence interval (CI) 29.7%–35.5%). Older age was strongly associated with prevalent HIV infection in bivariable analysis, as were a lower level of education, a higher level of income, and a higher number of lifetime pregnancies (Table 1). There were no significant differences in HIV prevalence between the five administrative areas in the city, by marital status, or by alcohol use. As expected, many self-reported sexual risk behaviors were associated with prevalent HIV, and condom use was protective (Table 2). Being a sex worker and having received payment for sex in the last 30 days were each associated with prevalent HIV infection but not significantly so (Tables 1 and 2). Having had two or more lifetime HIV tests or a primary partner who had been tested was associated with a lower HIV risk (Table 2). Women were asked whether their primary partner had ever been tested for HIV: those who answered ‘yes’ had the lowest risk of having prevalent HIV, and those who answered ‘don’t know’ had the highest risk (Table 2). Reporting at least one genital symptom in the last 3 months, a urinary tract infection (UTI) in the last year, or a gynecological pathology in the last year were each associated with prevalent HIV (Table 2), but having a positive pregnancy test at the study visit or ever having had a blood transfusion were not associated (data not shown).

In multivariable analysis, risk factors that remained statistically significant after controlling for other variables included: older age, lower educational level, self-reported genital symptoms in the last 3 months (adjusted odds ratio (aOR) = 1.4; 95% CI 1.1–2.0), having had more than one lifetime HIV test (aOR = 0.4; 95% CI 0.3–0.6), and not knowing whether the primary partner had ever tested for HIV (aOR = 1.7 when comparing to women who said that their primary partner had been tested for HIV at least once; CI 1.1–2.5) (Table 3).

**Table 3:** Risk factors associated with HIV infection in bivariable and multivariable analysis.

Characteristics	Crude OR* (95% CI)	Adjusted ORξ (95% CI)
**Age, years**	*P<0.001* § *P<0.001* ξ	*P<0.001* §
18–19	1.0	1.0
20–21	1.54 (0.98–2.41)	1.39 (0.86–2.25)
22–23	1.96 (1.23–3.11)	2.11 (1.29–3.45)
24–26	3.76 (2.40–5.89)	3.87 (2.38–6.29)
27–35	5.99 (3.78–9.49)	5.33 (3.67–8.44)
**Level of education**	*P<0.001* §	*P<0.001* ξ
None/primary	1.0	1.0
Incomplete secondary	0.48 (0.33–0.69)	0.64 (0.42–0.98)
Secondary and above	0.24 (0.15–0.37)	0.33 (0.20–0.54)
**Self-reported genital symptoms** 1 **last 3 mos. months monthsmonths** 1	*P = 0.043* *	*P = 0.013* ξ
No	1.0	1.0
At least 1 symptom	1.32 (1.01–1.72)	1.43 (1.06–1.95)
**HIV tests in lifetime** 2	*P<0.001* §	*P<0.001* ξ
Never	1.0	1.0
At least one	1.14 (0.84–1.52)	0.98 (0.70–1.39)
More than one (2–20)	0.55 (0.38–0.79)	0.41 (0.26–0.62)
**Primary partner ever tested for HIV** 3	*P<0.001* *	*P = 0.012* ξ
No	1.0	1.0
Yes	0.53 (0.36–0.77)	0.70 (0.46–1.06)
Do not know	1.18 (0.85–1.63)	1.22 (0.85–1.76)

OR: odds ratio. 95% CI: 95% Confidence interval. STI: sexual transmitted infection.

1 Genital symptoms: vaginal discharge (400), painful urination (133), lower abdominal pain (300), vaginal itching or burning (261), pain during intercourse (213), vaginal sore (54).

2 Missing data: number HIV test in lifetime = 1.

3 Not applicable (no PP) = 97. When comparing ‘Do not know’ to ‘Yes’ the aOR = 1.72 (1.12–2.45).

* Crude OR and P-value for the association between each variable and HIV-1 infection (chi-square test).

§ P-value from chi-square test for trend.

ξ OR and P-value adjusted for all variables in the table (likelihood ratio test).

### CD4 Count at HIV Diagnosis and Determinants of Late Diagnosis

**Figure 1:**
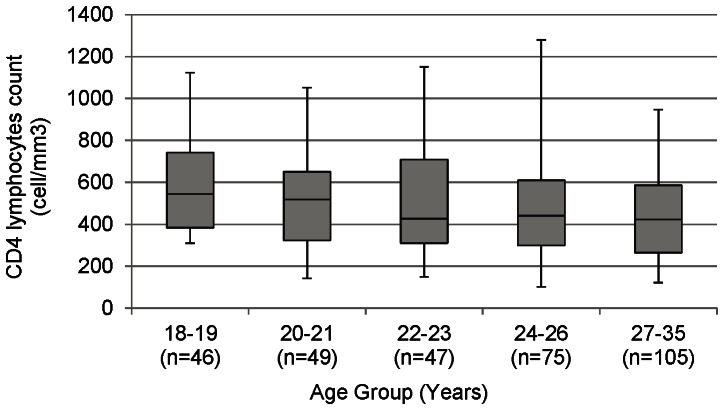
Median CD4 lymphocyte count among participants who tested HIV-positive.

Out of 332 women who had HIV-positive diagnoses, 96.9% had CD4 lymphocyte testing done on their HIV-positive whole blood sample sample (the rest of the CD4 count tests were invalid and could not be repeated). The median CD4 lymphocyte count for the HIV-positive women was 454 cells/*µ*L. Figure 1 illustrates the distribution of median CD4 lymphocyte counts and inter-quartile ranges across age groups. About a third (32.3%) of participants who tested HIV-positive in the study had a CD4 lymphocyte count ≤350 cells/*µ*L at diagnosis (defined as late diagnosis). Several factors were associated with late diagnosis in bivariable analysis including older age (p = 0.022), self-reported genital symptoms in the last 3 months (OR = 1.7; CI 1.0–3.0), having had a gynecological pathology in the last year (OR = 4.0; CI 1.3–12.5), and not knowing whether the primary partner ever tested for HIV (OR = 1.9; CI 1.0–3.6).

In multivariable analysis, not knowing whether the primary partner ever tested for HIV (aOR = 2.2; CI 1.1–4.2) and having had a gynecological pathology in the last year (aOR = 3.7; CI 1.2–12.0) remained statistically significantly associated with late diagnosis of HIV (Table 4).

**Table 4:** Risk factors associated with late HIV diagnosis (CD4≤350 cell/µL) in bivariable and multivariable analysis.

Characteristics	Crude OR* (95% CI)	Adjusted ORξ (95% CI)
**Primary partner ever tested for HIV**	*P = 0.007* *	*P = 0.007* ξ
No	1.0	1.0
Yes	0.87 (0.40–1.91)	0.94 (0.47–2.09)
Do not know	2.05 (1.13–3.73)	2.16 (1.13–4.16)
**Gynecological pathologies** 1	*P = 0.032* *	*P = 0.028* ξ
No	1.0	1.0
Yes	3.51 (1.12–11.02)	3.72 (1.15–12.01)

OR: odds ratio. 95% CI: Confidence Interval.

1 Gynecological pathologies: amenorrhea (1), dysmenorrhoea (1), genital warts (1), ovaries cysts (1), infertility (4), miscarriage (4), pelvic inflammatory disease (1), uterine myoma (1), vaginal bleeding (3).

* Crude OR for the association between each variable and CD4 count ≤350 cell/µL (chi-squared test).

§ P-value from chi-square test for trend.

ξ OR and P-value adjusted for all variables in the table (likelihood ratio test).

## Discussion

The current study shows that the HIV prevalence among women with two or more sexual partners in Beira, Mozambique (33%) is much higher than overall figures [3]. According to the 2009 national HIV prevalence survey, around 2% of women aged 15–49 years old in Sofala Province reported having two or more sexual partners in the past 12 months, with an HIV prevalence of 17.8% in this survey group. All of the women enrolled in our study were 18–35 years old and reported two or more sexual partners in the past one month The study sample represents a sizable group of women at higher risk of HIV acquisition, with an HIV prevalence of 32.6%, close to twice the provincial rate. The data obtained reflect our recruitment strategy targeting women at higher than average risk. It should be noted, however, that very few women self-identified as sex workers (3.4%); a much higher proportion of women reported having received payment for sex in the preceding month (18.4%).

Our study revealed a strong association between education and HIV infection, in accord with some but not all studies [13], [14]. It may be that women with higher education are better able to negotiate protected sex with sexual partners. Low-income levels were associated with increased risk of HIV infection in bivariable but not multivariable analysis. The national population-based HIV survey found that having some income (as opposed to no income or high income) was associated with HIV infection [4]. Our study did not find an association between HIV infection and marital status, which may be explained by the predominance of single women in the study. The existence of *lobola* (custom whereby the husband materially compensates the bride’s family for the bride’s hand in marriage) in Southern Africa tends to result in marriage at a later age than in other parts of Africa.

Self-reported genital symptoms in the last month were associated with HIV infection. This association suggests missed opportunities for earlier detection of HIV infections at local health post centers in Beira that provide clinical management of reproductive tract infections as well as HIV testing. It should be kept in mind that the medical factors captured in this study (such as gynecological pathologies) may be the result of HIV infection instead of actual risk factors for HIV.

Per World Health Organization (WHO) and Mozambique national treatment guidelines, one third (32.3%) of women were already eligible for HIV treatment at the time of diagnosis because their CD4 count was ≤350 cells/*µ*L [15], [16]. In addition, we found a strong protective effect of having had more than one lifetime HIV test, which is consistent with other studies in Mozambique [17]. Both of these findings highlight the importance of promoting regular HIV testing in HIV endemic areas, including Beira. HIV testing is free in Mozambique, yet substantial barriers to HIV testing remain such as poor transportation to health centers, test kit stock-outs and stigma associated with HIV testing and HIV infection. Other potential explanations include post-test changes in behaviors, or the possibility that many persons who seek testing are risk-averse. Participants who reported that they did not know whether their primary partner had ever been tested for HIV had a higher risk of HIV infection as well as late diagnosis of HIV in multivariable analyses. These findings contribute to the growing literature on how gender norms may influence sexual risk taking behaviors and risk reduction efforts in Mozambique [18]–[20] and suggests that HIV testing of couples might be an effective prevention strategy.

A strength of our study was that it was powered to explore possible risk factors for HIV infection with reasonable precision. However, the findings intentionally are not generalizable to all women in Beira, since the study population was skewed towards young women at higher risk for HIV. Even within our restricted population segment, the recruitment team may have missed some risk groups, such as bar workers since recruitment staff lacked incentives to work through the night and faced transportation and security challenges. Also, the brief standardized questionnaires could not delve into risk factors with depth, and may be prone to social desirability bias. Most important, causal inferences cannot be made based on the statistical associations found in this cross-sectional study.

Our collaborative team achieved the underlying objective to build novel HIV prevention research capacity in Beira, Mozambique. In so doing, we found a high prevalence of HIV infection among young women with two or more sexual partners, and frequent late diagnosis of HIV infection among them. Health authorities should strengthen HIV programs and strategies focusing on women at higher risk for HIV infection. If lower population viral load and reduced transmission are to be achieved, the problem of delayed testing and diagnosis needs to be addressed.
